# 头皮转移作为肺鳞癌的首发表现：1例极其罕见的病例报告及文献回顾

**DOI:** 10.3779/j.issn.1009-3419.2024.101.11

**Published:** 2024-04-20

**Authors:** Wenbo HE, Mingjun GAO, Qinglin REN, Mengmeng WANG, Siding ZHOU, Xiaolin WANG, Yusheng SHU

**Affiliations:** ^1^225000 扬州，扬州大学; ^1^Yangzhou University, Yangzhou 225000, China; ^2^116000 大连，大连医科大学; ^2^Dalian Medical University, Dalian 116000, China; ^3^225000 扬州，扬州大学附属苏北人民医院; ^3^Northern Jiangsu People's Hospital Affiliated to Yangzhou University, Yangzhou 225000, China

**Keywords:** 肺肿瘤, 转移, 皮肤肿瘤, 诊断技术与方法, Lung neoplasms, Metastasis, Skin neoplasms, Diagnostic techniques and procedures

## Abstract

原发性肺鳞癌远处转移中皮肤转移是一件罕有发生的事，而头皮转移作为患者首发的临床表现更加少见。头部皮肤转移灶很容易误诊为其他头部疾病，但它的出现提示着肺癌恶化与预后不良。这篇病例报告记录了1例以头部皮肤毛囊炎为首发表现就诊于皮肤科，通过影像学诊断为肺恶性肿瘤后转科至胸外科，头部病灶切除病理提示为肺癌的远处转移灶的女性患者，并对报道过类似病例的文章进行回顾总结。本文旨在增进对肺癌皮肤转移的了解和认识，提高对该类疾病的重视，加强早期识别、精准诊断，防止临床上对该疾病漏诊与误诊，最终达成推进后续的治疗、改善患者预后的目的。

## Abstract (en)

Distant cutaneous metastasis of primary lung squamous cell carcinoma is an exceedingly rare event, with scalp metastasis as the initial clinical presentation even rarer. Scalp skin metastases are prone to be misdiagnosed as other scalp disorders, yet their appearance signifies the deterioration and poor prognosis of lung cancer. This case report documents a female patient presenting initially with scalp folliculitis in dermatology, who was subsequently diagnosed with malignant lung tumor through radiological imaging and referred to Department of Thoracic Surgery. Pathological examination of the excised lesion from the scalp revealed distant metastasis of lung cancer. A review of similar cases reported in literature was conducted. This article aims to enhance understanding and awareness of skin metastasis in lung cancer, to emphasize the importance of this condition, and to improve early recognition and precise diagnosis. It is crucial to prevent clinical misdiagnosis and ensure appropriate treatment, finally leading to improve the prognosis of the patients.

肺癌发病率、死亡率居高不下，是癌症相关死亡的首要原因^[1]^，据国家中央癌症登记处统计2015年每天新诊断的肺癌病例平均超过2100例，约占所有癌症诊断的20%，同时，肺癌导致的每日平均死亡人数高达1700人，相当于所有癌症总死亡率的27%^[2]^。肺癌较常见的远处转移部位为脑、骨、肝和肾上腺等。皮肤转移在肺癌中的发生频率相对较低，晚期肺癌患者的皮肤转移率仅为2.8%^[3]^，然而，研究者们在探讨肺癌远处转移的研究中，得出的结果提示肺癌的头皮转移是一种极为罕见的临床表现。不同国家的研究^[4⇓-6]^均显示出现头皮转移临床表现的肺癌患者生存率极低，明确诊断后的生存期通常仅有2.9-4.9个月^[7]^。本文在介绍1例国内发生的肺癌头皮转移病例的同时，回顾并总结了其他相关文献对类似病例诊疗方式的描述。

## 1 病例报告

本文介绍了1例中年女性（51岁）肺癌头皮转移的病例，并对其诊疗过程进行了回顾与总结。患者最初因头部皮肤瘙痒伴局部疼痛就诊于皮肤科门诊，初步诊断为毛囊炎，未予特殊处理。数日后，患者因反复咳嗽咳痰再次就诊，胸部计算机断层扫描 （computed tomography, CT）（图1A）提示右肺下叶见软组织团块影，呈分叶状，较大层面大小约49 mm×32 mm，纵隔与肺门未见明显肿大淋巴结影，胸膜未见明显增厚。我院以“右肺下叶肺占位性病变”收治入院，患者否认高血压、糖尿病、冠心病及各类常见慢性病病史，否认结核病、肝炎、伤寒等传染病病史，否认常见食物药物过敏史及重大手术外伤史，患者入院后查肿瘤标志物六项示：结果显示糖链蛋白125为7.09 U/mL，甲胎蛋白为4.11 ng/mL，癌胚抗原为2.5 ng/mL，神经元特异性烯醇化酶为15.7 μg/mL，细胞角蛋白片段19（cytokeratin 19 fragment, CYFRA 21-1）为3.29 ng/mL，所有指标均处于正常值范围。在体格检查时，医生发现患者头部后方存在一个包块，为进一步明确包块的性质，遂决定进行头皮肿物活检。肉眼见：0.2 cm×0.2 cm×0.1 cm皮肤组织一块，切面灰白，质中，显微镜下观提示：鳞状上皮下见异性细胞呈巢状排列，浸润性生长，活检病理（图1B）提示：（头皮）转移或浸润性癌，结合临床病史符合来源于肺。免疫组化提示：CKpan（+）、CK20（小灶区+）、P63（+）、CK5/6（+）、Ki67（约10%+）、CD99（小灶区+），其余均为阴性。鉴于患者已出现远处转移，根据当前的临床评估，该情况不符合手术指征。因此，在与患者家属充分沟通后，家属决定采取保守治疗作为当前的治疗策略。患者出院后于外院查颅脑磁共振成像（magnetic resonance imaging, MRI）（图1C）提示：患者病灶累及皮下深部，较大截面约2.3 cm×2.0 cm，3个月后进行电话随访，患者明确病理为肺鳞状细胞癌（lung squamous cell carcinoma, LUSC）后已于外院积极行6个疗程化疗+3个疗程免疫治疗，皮下肿物行局部小剂量放疗，具体方案不详，患者一般状态良好。治疗后影像学见图1D、图1E，6个月后患者出现左上腹部转移灶，切除后病理（图1F）提示：肉眼见：1.5 cm×1.5 cm×1 cm结节1枚，切面灰白、质中，镜下见：异型细胞呈巢状排列，浸润性生长。于我院进行帕博利珠单抗200 mg单免疫治疗2次。随后，患者出现了肺部感染的症状，经临床评估，不能排除放射性肺炎的可能性。我们采取了积极的抗感染治疗措施，经过有效治疗，患者的病情得到控制，顺利出院。

**图 1:**
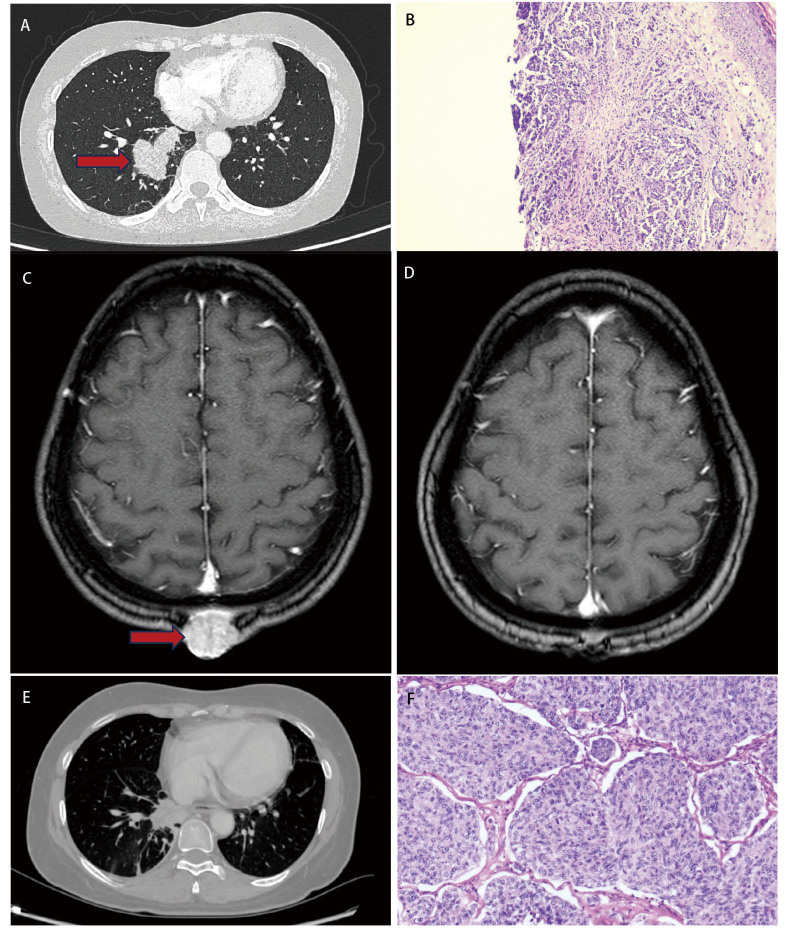
患者影像学表现及病理结果。A：治疗前右下肺占位性病变；B：头部皮肤肿块切除病理（HE染色，×40）；C：治疗前头皮转移病灶；D：头皮病灶予以局部小剂量放疗治疗后病灶明显缩小；E：4个疗程化疗后肺部病灶明显缩小；F：腹部包块切除病理（HE染色，×100）。

## 2 讨论

使用“肺鳞癌（Lung Carcinoma, Squamous Cell）”以及“皮肤转移（Cutaneous Metastasis）”作为关键词检索国内外相关病例报告共35篇，其中明确病理为LUSC且有较为详细的病例共21个，加上本篇报告共记录了22个病例（表1），其中18个病例为男性（81.82%），这与男性吸烟史致LUSC多发吻合。患者多为中老年（35-76岁），平均年龄为62.27岁。皮肤病变表现多为无痛性包块，共13例（56.52%），病灶多为单发（19例），这些临床表现主要取决于疾病诊断的早晚，6篇报道提出随着局部病变的发展，后续随访中皮肤病灶会在1-2个月内出现扩大与溃疡的表现。这些病例中仅3例对皮肤切除的组织进行了免疫组化分析，都提示有P63（+），其中有7例在切除局部病灶后，采用了全身化疗的治疗方式，除本例外，另外一篇报道的患者经治疗后也出现皮肤局部症状的较大缓解^[8]^。

**表 1:** 22例病例的临床特征

Item	Clinical characteristics
Gender	Male-female ratio: 4.5:1 (Male 18, Female 4)
Age	The average age was 62.27 years
Body region	7 cases of head and neck region (31.82%), 6 cases of multifocal involvement throughout the body (27.27%), 4 cases of abdominal region (18.18%), 4 cases of limb regions (18.18%), 1 case of thoracic region (4.55%)
Lesion type	13 cases of painless mass (59.09%), 5 cases of painless induration (22.73%), 3 cases of ulcerative nodule (13.64%), 1 case of erythema (4.55%)
Treatment	7 cases of systemic chemotherapy regimens (31.82%), 1 of them combined with a regimen of localized radiotherapy (4.55%)

正如本例所示，内脏转移瘤中，肺来源的转移在男性患者中占据显著比例，可高达24%，而在女性患者中则相对较少，仅占4%^[9]^，然而，尽管内脏皮肤转移瘤总体上在临床中较为罕见，头皮转移却在女性患者中显得相对更为常见^[10]^，这与头部皮肤丰富的血供有着较强关联，而转移至皮肤的肺癌组织学类型最常见的是腺癌，国内德吉央宗等^[11]^的研究中也报道了1例高级别胎儿型肺腺癌并头皮转移的患者，LUSC是第二最有可能发生转移的类型^[8]^。同时这些转移瘤的形态也多种多样，常以皮肤无痛性结节为主，也可发展为丘疹、斑块、溃疡、大疱、蜂窝织炎样病变或纤维化病变。这就给内脏皮肤肿瘤的诊断造成很多麻烦，可能被误诊为皮疹、皮下红斑、皮下硬结，甚至水肿、疝气等^[12]^，本例初诊时就被诊断为毛囊炎。

病理免疫组化结果可以为寻找原发灶提供有力的支持。对于诊断肺癌皮肤转移，特定的免疫组化标志物起着至关重要的作用^[13]^。例如：P63、抗甲状腺转录因子-1（thyroid transcription factor-1, TTF-1）、CK7和CK20^[14,15]^，其中P63（+）对于诊断LUSC转移有着较强的提示作用^[16]^，与此例的病理结果相符。Todisco等^[6]^的研究中提出可以使用下一代测序技术对皮肤病变与原发性肺结节的共同起源进行证实，CDKN2A和KRAS基因的致病突变也已经被多篇文献描述。皮肤远处转移通常是肺恶性肿瘤进展和预后不良的表现，其他不良预后指标包括小细胞原发性肺肿瘤（不可切除），多发性转移性皮肤病变，或其他远处转移，诊断为皮肤转移后的平均生存时间为2.9-4.9个月^[7,17]^。晚期肺癌转移的预后会受到原发灶的显著影响，因此主要的治疗方式还是对于原发灶的治疗^[3]^，针对一般情况稳定的患者，选择局部麻醉下手术切除皮肤转移灶已被研究证实为一种有效的治疗方式。这种手术方法旨在改善患者的生活质量，减少局部疼痛、出血等症状，并有望延长患者的生存期^[18]^。

总之，此类病例在临床上较为罕见，面对此类病例局部切除后病理检查是一种非常重要的诊断方式，一方面需要皮肤科医生在面对年龄较大、高癌症风险患者时提高警惕，及时取组织做病理活检以提高诊断成功概率；另一方面需要强调扩展多中心合作的必要性。我们希望通过此病例报道提高对此类罕见转移肿瘤的认识，为以后该类病例的分子诊断以及个性化治疗提供思路。
